# Analysis of factors related to tip malposition of peripherally inserted central catheter and its spontaneous correction rate in neonates

**DOI:** 10.4314/ahs.v25i2.15

**Published:** 2025-06

**Authors:** Lizhu Chen, Kunying Zhuang, Meili Zhang

**Affiliations:** Department of Neonatel Intensive Care Unit, Quanzhou Woman's and Children's Hospital, Quanzhou 362000, China

**Keywords:** Peripherally inserted central catheter, tip malposition, spontaneous correction, neonatal care

## Abstract

**Background:**

To analyze the factors associated with tip malposition and spontaneous correction rate of peripherally inserted central catheter (PICC) in newborns.

**Methods:**

Clinical data of 1604 newborns who underwent PICC placement in our neonatal surgery department from January 2018 to January 2023 were retrospectively analyzed. They were divided into malposition group (n=104) and normal group (n=1500) according to the occurrence of tip malposition. The relevant clinical data of newborns were extracted from the hospital information system. The occurrence of PICC tip malposition and spontaneous correction were recorded. Logistic regression analysis was used to identify risk factors for PICC tip malposition in newborns.

**Results:**

The incidence of PICC malposition was 6.48%. There were significant differences in gestational age, birth weight, lesion location, catheter direction and venous placement between the two groups (P<0.05). The independent risk factors for PICC tip malposition in newborns were lesion location, catheter direction and venous placement (P<0.05). The spontaneous correction rate within 24 hours was 27.88%, with the highest rate observed in malposition of the internal jugular vein.

**Conclusion:**

Catheter direction, lesion location and venous placement are independent risk factors for PICC tip malposition in newborns, and some newborns may spontaneously correct within 24 hours.

## Introduction

The blood vessels of newborns are characterized by thin walls and good permeability, which makes peripheral venous infusion difficult and prone to extravasation, increasing the risk of tissue necrosis. For critically ill newborns with a high demand for intravenous infusion, it is essential to establish a good venous access^1^. Currently, peripherally inserted central catheter (PICC) through peripheral vein puncture has been widely used in critically ill newborns, primarily to provide long-term effective venous access. PICC provides a reliable and long-term vascular access option, allowing for administration of fluids, medications, and parenteral nutrition. Its use in neonates offers several advantages, such as reduced need for multiple venipunctures, decreased discomfort, and improved patient safety. PICC can reduce puncture trauma and has a longer duration, which can better solve the problems of routine venous puncture in newborns^2^,^3^. However, PICC still has some drawbacks in practical application, such as catheter blockage or rupture, infection, catheter ectopia, embolism, etc. Among them, catheter tip ectopia is an important factor that can lead to a series of PICC-related complications, such as venous thrombosis, pleural effusion, and phlebitis, with an incidence rate of approximately 1.3% to 5%^4^,^5^. The Infusion Nurses Society INS - guidelines in the United States^6^ state that the safe position of the PICC catheter tip should be located at the lower 1/3 of the patient's superior vena cava or at the junction of the superior vena cava and right atrium. Therefore, tip ectopia of the PICC catheter usually refers to a position beyond the above safety location, such as the internal jugular vein or axillary vein, which is the most common. In clinical practice, catheter removal or partial withdrawal is often adopted for catheter tip ectopia. Severe cases require repositioning of the catheter. In actual clinical practice, some newborns experience spontaneous correction of PICC catheter ectopia, which means that the catheter tip moves to the upper or lower vena cava without any artificial intervention or external factors^7^,^8^. This spontaneous correction behavior not only reduces the risk of tip ectopia-related complications but also partially solves the problem of difficult repositioning of the catheter. This study analyzed the factors related to PICC catheter tip ectopia and the rate of spontaneous correction in our hospital's newborns, aiming to provide a basis for improving PICC.

## Patients and Methods

### Patients

A retrospective analysis was conducted on clinical data from 1,604 newborns who underwent peripherally inserted central catheter (PICC) placement in the neonatal surgery department of our hospital from January 2018 to January 2023. Among them, 104 cases experienced PICC tip malposition, while the remaining 1,500 cases did not. Based on this, the newborns were divided into the malposition group (n=104) and the normal group (n=1500).

**Inclusion criteria:** 1. Only include patients within the defined age range for newborns; 2 Include only newborns who underwent PICC placement and exclude other types of vascular catheter insertions; 3. Include patients with complete and reliable clinical data records to ensure data accuracy and analyzability; 4. Include newborns who underwent PICC placement within the specified time range to ensure data consistency and relevance.

**Exclusion criteria:** 1. Exclude newborns with severe complications that may significantly affect the positioning and outcomes of the PICC; 2. Exclude newborns who underwent simultaneous placement of other types of vascular catheters to ensure result accuracy and consistency; 3. Exclude patients with missing key data (e.g., PICC insertion site, catheter size, duration of catheterization, etc.) to ensure the integrity of the analysis.

### Study methods

#### Clinical data

Relevant clinical data of the newborns were extracted from the hospital information system. Data collection included demographic information, medical history, indications for PICC placement, PICC insertion site, catheter size, duration of catheterization, and complications.

#### PICC tip malposition rate

According to the US INS guidelines^6^, bedside X-rays were taken immediately after puncture to determine if the catheter tip was not in the target range of the fourth to sixth thoracic vertebrae, indicating malposition.

#### Univariate analysis of PICC tip malposition

Factors related to PICC tip malposition, such as gestational age, sex, birth weight, days of catheterization, direction of catheter placement, site of lesion and vein of catheter placement, were included in the analysis.

#### Spontaneous correction of PICC tip malposition

Bedside X-rays were taken again 24 hours after catheterization for newborns with good blood return but PICC tip malposition to observe spontaneous correction and calculate the rate of spontaneous correction.

### Statistical analysis

Data analysis was performed using Statistical Package for the Social Sciences (SPSS) 21.0 statistical software (IBM, Armonk, NY, USA). Rates were used to express count data, and the chi-square test was used. Normally distributed measurement data were expressed as mean ± standard deviation, and the t-test was used to compare intergroup differences between the malposition and normal groups. Logistic multiple regression analysis was used to identify risk factors for PICC tip malposition in newborns. A significance level of α=0.05 was used for two-tailed tests, and P<0.05 indicated statistical significance.

## Results

### Clinical data comparison between the eczema group and the normal group of newborns

In the eczema group, there were 44 male and 60 female newborns, with a gestational age at birth of (37.04±2.57) weeks and a birth weight of (2.51±0.53) kg. The age at catheterization was (13.16±3.25) days. In the normal group, there were 763 male and 737 female newborns, with a gestational age at birth of (38.25±3.35) weeks and a birth weight of (2.72±0.59) kg. The age at catheterization was (12.90±3.19) days. There were no statistically significant differences in sex and age at catheterization between the two groups (P>0.05). There were statistically significant differences in birth weight and gestational age at birth between the two groups (P<0.05).

### Incidence of peripherally inserted central catheter (PICC) malposition in newborns

Among the 1604 newborns, 104 cases had malposition of the PICC tip, and the remaining 1500 cases did not. The incidence of PICC malposition was 6.48%.

### Univariate analysis of neonatal PICC tip malposition

Univariate analysis of newborns in the two groups showed no significant difference in gender or age at catheterization (P > 0.05). There were significant differences in gestational age, birth weight, lesion site, catheterization direction and catheterization vein (P < 0.05). See Table 1.

**Table 1:** Univariate analysis of neonatal PICC tip heterotopia (*x̅*±*s*)

Category	Ectopic group (*n* = 104)	Normal group (*n* = 1500)	*T/χ* ^2^	*P*
Gestational age			11.020	0.001
Premature delivery	28 (26.92)	221 (14.73)		
Term	76 (73.08)	1279 (85.27)		
Gender			2.850	0.091
Male	44 (42.31)	763 (50.87)		
Female	60 (57.69)	737 (49.13)		
Birth weight			30.221	0.000
Normal body mass	55 (52.88)	1153 (76.87)		
Low birth weight	46 (44.23)	322 (21.47)		
Very low birth weight	3 (2.88)	25 (1.67)		
Age at catheterization (days)			1.895	0.594
1 week after birth	64 (61.54)	918 (61.20)		
2 weeks after birth	25 (24.04)	324 (21.60)		
3 weeks after birth	10 (9.62)	205 (13.67)		
4 weeks after birth	5 (4.81)	53 (3.53)		
Insertion direction			4.688	0.030
LEFT	63 (60.58)	744 (49.60)		
Right	41 (39.42)	756 (50.40)		
Lesion site			23.617	0.000
Thorax	67 (64.42)	603 (40.20)		
Abdomen	31 (29.81)	722 (48.13)		
Other	6 (5.77)	175 (11.67)		
Catheterized vein			50.157	0.000
Basilic vein	48 (46.15)	1121 (74.73)		
Superficial temporal vein	36 (34.62)	379 (19.80)		
Other	20 (19.23)	82 (5.47)		

Note: Term refers to gestational age of 37 weeks, premature delivery refers to gestational age of 38 weeks but less than 37 weeks; normal body weight is 2.5 ∼ 4.0 kg, low birth weight is 1.5 kg ∼ 2.5 kg, very low birth weight is less than 1.5 kg.

### Multivariate Analysis of Neonatal PICC Tip Ectopy

Multivariate analysis showed that the independent risk factors of neonatal PICC tip ectopy were lesion location, catheterization direction and catheterization vein (P < 0.05). See Tables 2 and 3.

**Table 2:** Variable Assignment

Variable	Assignment value
PICC tip malposition	Normal = 0, Ectopic = 1
Gestational age	Term = 0, preterm = 1
Birth weight	Normal weight = 1, low birth weight = 2, very low birth weight = 3
Lesion site	Chest = 1, Abdomen = 2, Other = 3
Insertion direction	Right = 0, Left = 1
Catheterized vein	Basilic = 1, Superficial temporal = 2, Other = 3

**Table 3:** Multivariate analysis of neonatal PICC heterotopia

Independent variable	*B*	*SE*	Wald *χ*^2^	*OR*	*P*	95% *CI*
Gestational age	0.527	0.419	1.582	1.694	0.209	(0.745, 3.851)
Birth weight	0.574	0.373	2.368	1.775	0.125	(0.855, 3.688)
Lesion site	-0.616	0.247	6.220	0.540	0.013	(0.333, 0.876)
Insertion direction	0.878	0.3	6.152	2.406	0.014	(1.202, 4.816)
Catheterized vein	0.950	0.331	8.237	2.586	0.004	(1.325, 4.947)

### Spontaneous Correction of PICC Ectopy in Neonates

Among 104 newborns with PICC tip heterotopia, 29 newborns had spontaneous correction of PICC tip heterotopia, of which 4 had too deep position after reduction and withdrew part of the catheter, respectively. Spontaneous correction rate of newborns at 24 hours was 27.88%. PICC tip heterotopia was different, and the ectopic site with the highest spontaneous correction rate was the internal jugular vein, which was 69.44%.

## Discussion

PICC provides a long-term venous access that effectively reduces the pain caused by repeated puncture processes, saves the time and energy cost of medical staff, and ensures the venous nutritional supply of newborns^9^. However, in clinical practice, PICC placement is not without flaws, and there are many issues surrounding its actual application, of which tip malposition is the most common^10^. Tip malposition caused by any reason will have a significant impact on newborns. Due to the difficulty of controlling newborn limb movements, the rapid increase in body length and weight, and the shortness of the superior vena cava, PICC placement is difficult and management is complicated. Although some infants with tp malposition have spontaneous correction behavior, tip malposition is still an important issue in neonatology that cannot be ignored.

The results of this study showed that 6.48% of the 1604 newborns with PICC placement had tip malposition. Foreign studies have shown^11^-^14^ that the rate of tip malposition is about 8.1% to 56.0%, which is somewhat different from the results of this study. This may be because this study mainly selected newborns with upper limb venous catheterization as the research object, and did not statistically analyze and study the incidence of malposition in lower limb venous catheterization.

In clinical practice, tip malposition of PICC in newborns is influenced by many factors. When tip malposition occurs in non-central veins or the right atrium, a series of complications can be caused. Foreign studies have reported that the incidence of newborn chest or pericardial effusion caused by PICC malposition is 0.05% to 0.76%, which seriously threatens the quality of life and safety of newborns^15^-^17^. Therefore, analyzing the factors related to tip malposition of PICC in newborns has important clinical significance and can guide more scientific interventions in clinical practice.

The results of this study showed that there were differences in gestational age, birth weight, lesion location, catheterization direction, and catheterization vein between the ectopic group and the normal group of newborns. Further analysis using logistic regression model with statistically significant single-factor independent variables revealed that the independent risk factors for ectopic PICC tip in newborns were lesion location, catheterization direction, and catheterization vein.

Peripheral veins in newborns are characterized by their small size, shallow location, and numerous branches, making PICC catheterization more difficult. A one-time placement of the PICC tip at the target location is challenging. Choosing larger, straighter, and less valvular veins during catheterization can reduce the incidence of ectopic PICC tips. Therefore, veins such as the cephalic vein and the basilic vein, which are shallow and have larger and straighter blood vessels, are more suitable for puncture, and the incidence of ectopic PICC placement is lower. This study also showed that the proportion of newborns in the normal group who underwent catheterization through the cephalic vein was significantly higher, indicating that the catheterization vein is an important influencing factor for ectopic PICC tip. In addition to choosing veins that are easy to puncture, the specific puncture vein for newborns should be considered based on factors such as the surgical site.

The superior vena cava receives the blood from the cephalic and superficial temporal veins, and most newborns who require PICC placement have congenital malformations, which increases the likelihood of vascular malformations, which directly affect the direction of blood vessels in the catheterization area. Since the subjects of this study were newborns who underwent upper limb vein catheterization, the incidence of ectopic PICC tips in newborns with chest lesions would be higher. The results of this study also showed that the ectopic group of newborns had lesions primarily in the chest area.

Based on neonatal anatomy, the distance from the right upper limb vein to the superior vena cava is shorter than that of the left, with fewer venous valves, providing a natural advantage for puncture. Although the majority of the Chinese population is right-handed, for newborns, the impact of anatomical structure on catheter misplacement is significant. In this study, most of the misplacements occurred in newborns who underwent catheter placement through the left upper limb, indicating that the direction of catheter placement affects the rate of PICC tip misplacement.

Factors such as hemodynamics, limb movement, and changes in posture can promote the movement of soft tissues around the catheter, changing the direction of the blood vessels and causing the position of the catheter tip to change. This can result in the catheter tip being adjusted towards the heart, and even being repositioned to the optimal target location. The results of this study showed that among 104 newborns with PICC tip misplacement, 27.88% spontaneously self-corrected within 24 hours, with the highest spontaneous correction rate occurring in the internal jugular vein. Previous studies by Rastogi and others have indicated that the spontaneous correction rate is higher when the PICC tip is misplaced in larger blood vessels. Tawil and others have further demonstrated that all newborns with PICC tip misplacement in large venous vessels self-corrected within 24 hours of catheter placement.

For newborns with PICC tip misplacement, manual adjustment, passive limb movement, and physiological saline infusion can be used to promote catheter tip repositioning. It is not recommended to immediately remove or withdraw the catheter for newborns who show catheter tip misplacement on their first X-ray. Instead, imaging results after 24 hours can be used to determine whether repositioning is necessary. For newborns who have not repositioned, reconsideration of catheter placement or removal can be considered. Throughout the process, continuous electrocardiographic monitoring should be performed on newborns who undergo PICC catheter placement, and their condition should be closely observed to promptly take effective measures to prevent unnecessary adverse events.

The limitations of this study are as follows: This study was conducted retrospectively at a single center, which may limit the generalizability of the findings to other clinical settings or populations; The study did not consider certain factors that could influence PICC tip malposition, such as operator experience, catheter insertion technique, or use of imaging guidance;This study focused on identifying risk factors and analyzing immediate outcomes related to PICC tip malposition. Long-term follow-up data on the clinical consequences or complications associated witmalposition were not included.

## Conclusion

In summary, the main influencing factors for the malposition of the tip of a peripherally inserted central catheter (PICC) in newborns are the direction of placement, the location of the lesion, and the placement vein. While spontaneous correction may occur within 24 hours in some newborns, additional intervention is necessary for the majority of cases. It is important to note that this study only considered PICC placement via the upper limb veins and did not take into account the occurrence and spontaneous correction of malposition in the lower limb veins. This limitation may introduce bias into the results. Additionally, the selection of only representative and important veins, such as the basilic vein and cephalic vein, as the primary puncture targets also limits the generalizability of the study to other venous access sites.

## Figures and Tables

**Table 4:** Spontaneous Correction of PICC Ectopy in Neonates (n, %)

Ectopic site	Ectopic cases	Spontaneous correction	Spontaneous correction rate
Internal jugular vein	36	25	69.44
External jugular vein	22	2	9.09
Submental vein	17	1	5.88
Cephalic vein	6	0	0.00
Axillary vein	9	0	0.00
Subclavian vein	14	1	7.14
